# Conservative Management of Presumptive Free Flap Pedicle Compromise on Postoperative Day 6: Report of Two Cases and Literature Review

**DOI:** 10.1002/micr.70314

**Published:** 2026-10-31

**Authors:** Filippo A. G. Perozzo, Diwakar Phuyal, Julia Ting, James Owens Gaston, L. Scott Levin, Alberto Franchi

**Affiliations:** ^1^ Division of Plastic Surgery, Department of Surgery University of Pennsylvania Philadelphia Pennsylvania USA; ^2^ Department of Orthopaedic Surgery Penn Medicine University City Philadelphia Pennsylvania USA; ^3^ Department of Plastic and Reconstructive Surgery Cleveland Clinic Foundation Cleveland Ohio USA; ^4^ Case Western Reserve University School of Medicine Cleveland Ohio USA; ^5^ Department of Hand and Plastic Surgery Cantonal Hospital of Winterthur Winterthur Switzerland

**Keywords:** conservative management, flap inosculation, free tissue transfer, neovascularization, pedicle compromise

## Abstract

Pedicle compromise after free tissue transfer typically mandates urgent surgical re‐exploration, as flap viability is considered entirely dependent on an intact vascular supply before inosculation is achieved. Whether selective conservative management can be considered in selected cases remains an open question. We report two cases of lower extremity free flap reconstruction in which presumptive late pedicle compromise—occurring on postoperative day 6 in both patients—was managed conservatively with full flap survival. Case 1 was a 71‐year‐old non‐smoking woman with hypertension who underwent coverage of a traumatic pretibial bone‐exposing defect (measuring approximately 9 × 5 cm) with a free superficial circumflex iliac artery perforator (SCIP) flap (approximately 10 × 6 cm) anastomosed to the posterior tibial perforator and a deep vein. Case 2 was a 33‐year‐old moderate smoker who underwent deep inferior epigastric perforator (DIEP) flap reconstruction (approximately 15 × 9 cm) for tibial plate exposure following a fracture fixation and wound dehiscence, anastomosed to the posterior tibial perforator and deep vein. In both cases, loss of Doppler signal and clinical ischemia on postoperative day 6 were accompanied by bedside indocyanine green (ICG) imaging demonstrating peripheral perfusion originating from the flap margins distant from the anastomosis, consistent with early neovascularization. Re‐exploration was deferred in both cases. Both flaps developed minor marginal epidermal loss without full‐thickness necrosis, and achieved complete healing within 6 weeks. Follow‐up was 6 months in both cases. These cases suggest that, in carefully selected patients with multimodal evidence of peripheral flap perfusion, conservative management of presumptive late pedicle compromise may be considered. The accompanying literature review is subject to publication bias, as only cases with successful outcomes are systematically reported; the true failure rate of conservative management cannot be estimated from available evidence. These findings are hypothesis‐generating and should not be interpreted as a new standard of care; prospective validation is required before definitive recommendations can be made.

## 1 Introduction

Free tissue transfer is a mainstay of reconstructive surgery, with flap survival traditionally considered dependent on an intact vascular pedicle. Early pedicle compromise—conventionally defined as thrombosis occurring within 72 h of surgery—is typically managed with urgent re‐exploration, as it is considered a critical threat to flap viability (Kroll et al. 1996; Bui et al. 2007), occurring before the flap has achieved sufficient inosculation (Mücke et al. 2012; Granzow et al. 2015; Bradshaw and Wagels 2017; Ludolph, Cai, et al. 2019; Kumar et al. 2004). Throughout this report three related but distinct terms are used consistently: neovascularization (used synonymously with angiogenesis) denotes the ingrowth of new vessels from the recipient bed and wound margins into the flap; inosculation denotes the direct linkage of these ingrowing vessels with the flap's own subdermal vasculature; and autonomization denotes the resulting functional state in which flap perfusion becomes independent of the original vascular pedicle (Yoon and Jones 2016).

Evidence from experimental and clinical studies suggests that neovascularization from the recipient bed can begin within days after flap transfer, potentially sustaining tissue even if the pedicle fails (Yoon and Jones 2016; Velander 1964; Oswald et al. 1988; Black et al. 1978). Yet, the timing and reliability of this process remain variable and are influenced by factors such as flap composition, recipient site quality, patient comorbidities, and prior radiotherapy (Yoon and Jones 2016; Salgado et al. 2002; Clarke et al. 1985; Cordeiro et al. 1999). This variability creates uncertainty in clinical decision‐making when pedicle compromise arises beyond the first 72 h (late compromise—events beyond 72 h postoperatively, a period when partial flap autonomization may already be underway), since autonomization may not yet be established enough to sustain tissue survival independent of the pedicle.

In this context, we present two cases of free flaps with presumptive late pedicle compromise on postoperative day 6 that ultimately remained viable without re‐exploration. Motivated by these observations, we reviewed the existing literature on free flap survival despite documented pedicle compromise, with the aim of identifying factors associated with flap survival and exploring whether selective conservative management can be safely considered. Late pedicle compromise and pedicle‐independent flap survival have been reported before. What these cases add is a specific combination absent from earlier reports: survival documented at postoperative day 6, one of the earliest timepoints in the published literature; both recipient sites in the lower extremity, a setting that accounts for fewer than half of reported cases and carries distinct challenges (dependent positioning, reduced soft tissue vascularity, limited vessel caliber) not encountered in head and neck reconstruction; and a conservative decision guided in real time by bedside indocyanine green (ICG) angiography using the Fluobeam system, an application not previously described in detail in this context. The two cases are presented together because they share the same timing, recipient site, management approach, and monitoring tool; each corroborates what the other suggests.

## 2 Case Reports

### 2.1 Case 1

A 71‐year‐old female patient, non‐smoker with a history of hypertension, presented with a flap wound at the upper base of the left pretibial region following a fall while boarding a bus. The wound, measuring approximately 9 × 5 cm with tibial bone exposure, was initially managed conservatively; however, progressive demarcation of necrosis necessitated surgical reconstruction (Figure 1a). A free superficial circumflex iliac artery perforator (SCIP) flap (approximately 10 × 6 cm) was harvested from the left inguinal region. A posterior tibial artery perforator and a deep vein were isolated in the leg, and arterial and venous end‐to‐end anastomoses were performed using coupling devices (Figure 1b). Immediate postoperative ICG angiography confirmed satisfactory anterograde perfusion from the anastomotic side (Figure 1c).

**FIGURE 1:**
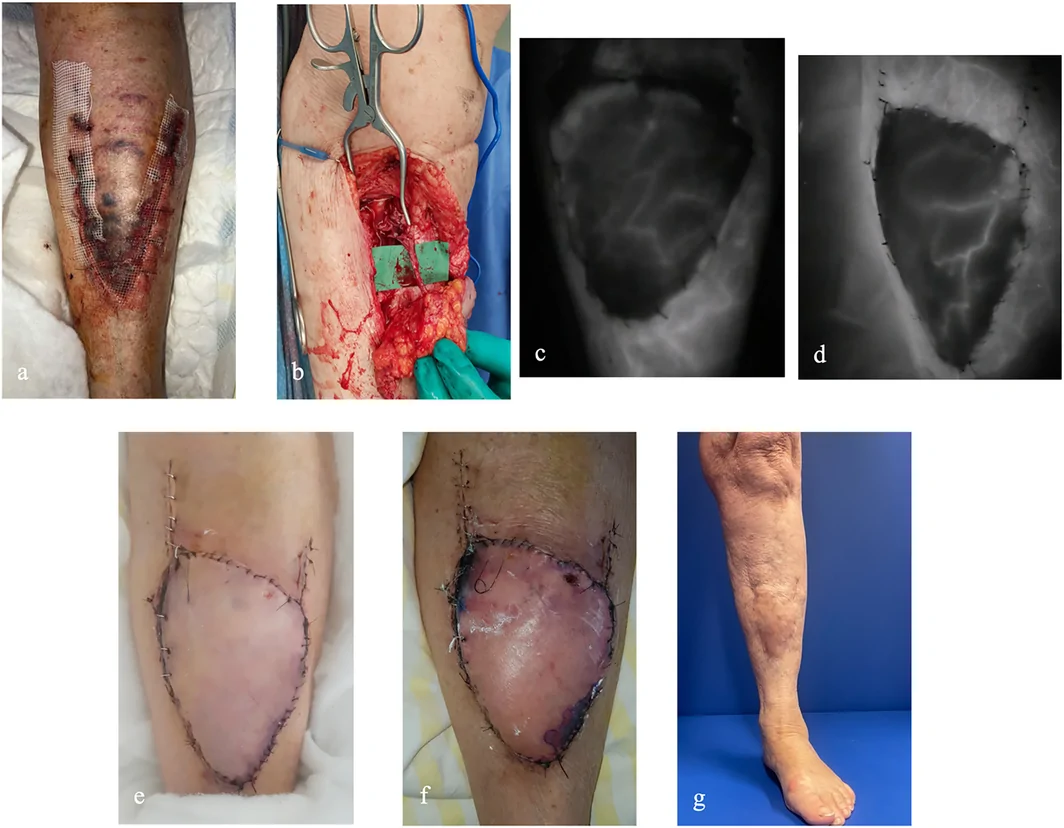
Case 1—Free SCIP flap reconstruction for a pretibial defect. (a) Preoperative wound with tibial bone exposure. (b) Intraoperative view showing flap inset and vascular anastomoses to the posterior tibial perforator and deep vein. (c) Early postoperative ICG dynamic angiography demonstrating anterograde vascular flow from the anastomotic side, with the pedicle entry point and its perfusion territory clearly visible. (d) Postoperative day 6 ICG dynamic angiography after loss of Doppler signal, showing peripheral perfusion originating from the flap margin opposite the anastomosis; the vessel opacified runs in the opposite anatomical direction to the pedicle seen in (c), excluding retrograde pedicle flow. (e) Clinical appearance on postoperative day 6 at the time of complete Doppler signal loss, showing diffuse pallor and absent capillary refill consistent with flap ischemia. (f) Day 10 postoperative evaluation with marginal epidermal necrosis but preserved central flap viability. (g) Complete flap healing two months after surgery.

Postoperatively, the flap was monitored hourly for the first three days and every three hours thereafter, with handheld Doppler recordings at the anastomotic site. On the sixth postoperative day, the Doppler signal was no longer detectable. The patient had ambulated on crutches with the limb dependent. The flap was diffusely pale with absent capillary refill, consistent with overt ischemia (Figure 1e). The diagnosis of pedicle compromise rested on two concurrent clinical criteria, independent of imaging: complete loss of Doppler signal at the anastomotic site and overt clinical ischemia. Angiographic confirmation of thrombosis was not obtained; the diagnosis therefore remains presumptive. Bedside Fluobeam ICG dynamic angiography was performed. The day‐6 sequence showed peripheral filling from the contra‐pedicle margin (Figure 1d). Compared with the early postoperative sequence (Figure 1c), the vessel opacified on day 6 courses away from the pedicle axis, excluding true retrograde pedicle flow, which would propagate back along that axis. Retrograde filling occurs when collateral or neovascular channels perfuse a vessel from its distal end in the absence of dominant antegrade pedicle inflow—a recognized consequence of peripheral neovascularization that can sustain flap viability after pedicle occlusion. The pattern observed here is consistent with this mechanism: peripheral ingrowth from the contra‐pedicle margin with no backpropagation along the pedicle vessel. The decision to observe was based on these two clinical criteria together with the ICG pattern, which collectively argued against functioning anterograde pedicle flow. The pattern was stable on all subsequent assessments. Given the time elapsed and the multimodal evidence of peripheral perfusion, re‐exploration was not pursued. Minor epidermal distress was noted at the flap borders on day 10 (Figure 1f), but no full‐thickness necrosis developed. The flap recovered its color and pigmentation and healed completely by two months (Figure 1g).

### 2.2 Case 2

A 33‐year‐old female, a moderate smoker with no other comorbidities, was referred for reconstruction of surgical wound dehiscence and tibial plate exposure following internal fixation of a left tibia fracture (Figure 2a). A thin deep inferior epigastric perforator (DIEP) flap (approximately 15 × 9 cm) was selected to utilize abdominal skin redundancy. Arterial anastomosis was performed to a posterior tibial artery perforator and venous anastomosis was completed using a coupler to a deep vein (Figure 2b). Flap perfusion was documented hourly for the first 48 h and every three hours until the fifth postoperative day, with all clinical and Doppler assessments remaining satisfactory throughout. During the night of postoperative day 5, the patient briefly ambulated on crutches. By the following morning (postoperative day 6), the flap appeared clinically pale with diffuse pallor, early marbling, and absent capillary refill—findings consistent with overt ischemia (Figure 2c). Importantly, the peripheral margins of the flap—specifically those in direct contact with the surrounding wound bed and healthy subcutaneous tissue—retained a pinkish hue, contrasting with the central pallor. This differential appearance indicated that peripheral vascular connections with the surrounding healthy tissue had already formed, providing an independent route of perfusion at the flap‐wound interface independent of the pedicle. Handheld Doppler assessment was performed systematically along the pedicle course: signal was absent at the anastomotic site, absent at every previously marked monitoring point between the anastomosis and the flap, and absent at the pedicle entry into the flap itself. Only faint, non‐pulsatile signals were detectable at the distal flap periphery, well beyond the pedicle territory, consistent with marginal perfusion from the surrounding wound bed rather than residual pedicle‐derived inflow. The diagnosis of pedicle compromise was based on these two concurrent clinical criteria—complete absence of Doppler signal along the pedicle axis and overt clinical ischemia—established independently of ICG imaging. Bedside Fluobeam monitoring was subsequently performed and demonstrated ICG fluorescence signal at the flap margins, distant from the pedicle insertion, consistent with early peripheral neovascularization. The original ICG image files were not retained on the device at the time of assessment and are therefore unavailable for inclusion; this is a recognized limitation of this case. The management decision rested primarily on the full clinical picture: central ischemia with overt pallor alongside viable pink peripheral margins, peripheral Doppler preservation, and 6 days of survival without full‐thickness necrosis—together indicating sufficient peripheral autonomization to sustain the flap without pedicle‐based inflow. The ICG fluorescence was consistent with that assessment but was not the sole basis for it. Surgical re‐exploration was deferred on the basis of the full clinical picture. As in Case 1, definitive confirmation of pedicle thrombosis was not obtained; the diagnosis of pedicle compromise in this case therefore remains presumptive. Over the following days, the flap developed minor epidermal loss without full‐thickness defects (Figure 2d). Complete healing was achieved with regular wound dressings after discharge (Figure 2e). Follow‐up at 1 month confirmed a well‐integrated flap with no additional surgical intervention required. At 6 months, the flap remained fully viable with no wound complications.

**FIGURE 2:**
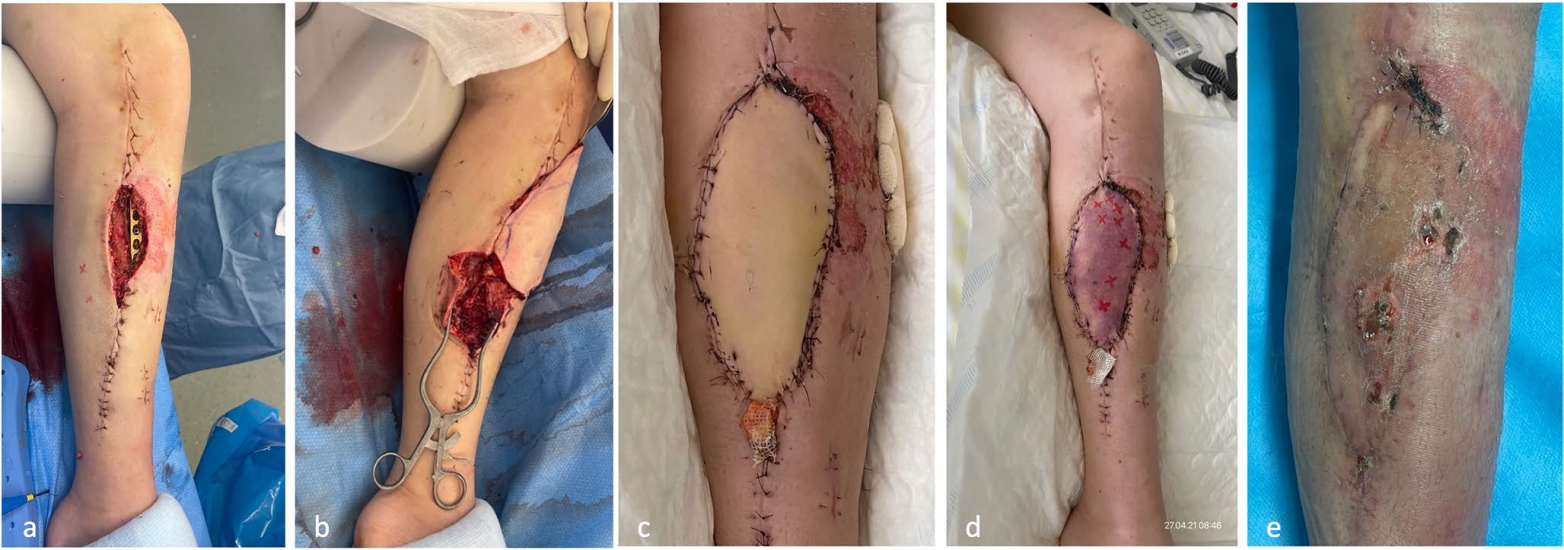
Case 2—deep inferior epigastric perforator (DIEP) flap reconstruction for a left tibial soft tissue defect with exposed osteosynthesis material. (a) Preoperative wound after debridement showing plate exposure. (b) Intraoperative view of thin DIEP flap with arterial and venous anastomoses to the posterior tibial perforator and deep vein. (c) Flap ischemia observed on postoperative day 6 following patient mobilization. (d) Flap appearance on postoperative day 10, showing persistent viability. (e) Nearly complete flap healing 1 month after surgery.

## 3 Discussion

The two cases presented here illustrate a clinical scenario that is both uncommon and underreported: free flap survival despite presumptive late loss of pedicle perfusion on postoperative day 6, without surgical re‐exploration. Together, they prompt a review of the existing literature on pedicle‐independent flap survival and raise questions about the conditions under which conservative management may be appropriate. Late pedicle compromise and pedicle‐independent flap survival have been reported previously; what distinguishes these cases is their combination of unusually early timing, recipient site, and monitoring approach. Pedicle‐independent survival at postoperative day 6 is among the earliest described in the clinical literature; the lower extremity setting introduces challenges—dependent positioning, reduced soft tissue vascularity, limited vessel caliber—that are not encountered in head and neck cases, which make up over half of published reports, and the prospective use of bedside real‐time ICG angiography to guide a conservative decision in this specific context has not been previously described in detail. These features, taken together, represent the specific contribution of this report to an otherwise well‐established body of literature.

Free flaps initially depend entirely on their vascular pedicle for perfusion, and arterial or venous compromise within the first 48–72 h typically leads to flap failure (Kroll et al. 1996; Bui et al. 2007). However, experimental and clinical evidence demonstrates that neovascularization and angiogenesis from the recipient bed can begin within the first postoperative week, allowing some flaps to survive early vascular compromise. Venous neovascularization may start as early as postoperative day 2, while arterial inflow from surrounding tissues can sustain flap viability even if the pedicle fails, demonstrating that early survival is biologically plausible under favorable conditions (Yoon and Jones 2016; Velander 1964; Oswald et al. 1988; Black et al. 1978; Tsur et al. 1980; Ceulemans et al. 2001).

The timeline and extent of this process are influenced by several variables, including flap tissue composition, volume, recipient bed quality, prior radiation, and patient characteristics (Yoon and Jones 2016; Salgado et al. 2002). Cutaneous and fasciocutaneous flaps tend to undergo autonomization faster than muscle or jejunal flaps, while larger or osteocutaneous flaps may require longer for complete perfusion (Clarke et al. 1985; Cordeiro et al. 1999). Preoperative radiotherapy delays but does not completely prevent neovascularization (Clarke et al. 1985; Schultze‐Mosgau et al. 2002).

A systematic search of PubMed, Embase, and Scopus was conducted using keywords and Medical Subject Headings (MeSH) terms related to free flap survival, vascular compromise, flap autonomization, and neovascularization. The review was conducted and reported in accordance with the Preferred Reporting Items for Systematic Reviews and Meta‐Analyses (PRISMA) 2020 statement. Each database was searched from inception to 31 October 2025, with no start‐date restriction; the strategy combined controlled vocabulary (MeSH and Emtree) with free‐text synonyms for free or microvascular flaps, pedicle or anastomotic thrombosis and vascular compromise, flap salvage, autonomization, inosculation, and neovascularization, linked with the Boolean operators AND and OR and adapted to the syntax of each database. Reference lists of all included articles and of relevant reviews were hand‐searched for additional eligible cases, and duplicate records were removed before screening. English‐language case reports and series reporting individual patient data were included if arterial (±venous) pedicle compromise occurred after postoperative day 3 and the flap survived without successful re‐anastomosis. Excluded were studies lacking case‐level data, complete flap necrosis, intentional pedicle division, successful re‐anastomosis, and animal or basic science studies. After screening 2357 records and reviewing 100 full texts, 18 publications describing 29 cases were identified. Titles and abstracts were screened independently by two reviewers, and the full texts of potentially eligible records were then assessed against the same criteria; disagreements at either stage were resolved by discussion and, where consensus could not be reached, by adjudication from the senior author. Data were extracted independently by two reviewers using a standardized, piloted extraction form and were cross‐checked against the source article, with any discrepancies reconciled by consensus. For each case the following variables were recorded where reported: patient age and sex, comorbidities and smoking status, flap type and tissue composition, recipient site, the postoperative day and mode of pedicle compromise, the monitoring and diagnostic modalities employed, the management strategy adopted (conservative observation versus operative re‐exploration), and flap outcome with duration of follow‐up. A PRISMA flowchart is provided as Figure S1. This review was not prospectively registered; given its retrospective and exploratory nature within a case series, formal PROSPERO registration was not obtained. Risk of bias was not assessed with standardized tools (such as RoB 2 or ROBINS‐I), as all included studies are case reports or small series for which such instruments are not designed. Case‐level data quality was instead evaluated by assessing the presence of key variables: patient demographics, precise timing of pedicle compromise, monitoring methods, and documented outcome. It must be acknowledged at the outset that this literature is subject to publication bias: the 29 identified cases represent exclusively successful outcomes, and cases in which conservative management was attempted but the flap was lost are systematically absent from the published literature. This absence of failure data is the most important limitation of the entire evidence base and must be borne in mind throughout the following analysis.

The 29 cases (median patient age 59 years, interquartile range (IQR) 38–68; 62.1% male) included a range of flap types: fasciocutaneous (34.5%), osteocutaneous (20.7%), musculocutaneous or muscle with split‐thickness skin graft (20.6%), replantation or free digit transfer (10.3%), jejunal (10.3%), and muscle only (3.4%). Recipient sites were predominantly head and neck (51.7%) and extremities (41.4%), with breast reconstruction in 6.9%. Preoperative radiotherapy was reported in 17.2% of cases. The postoperative day (POD) of definitive pedicle compromise, used as a proxy for flap autonomization, had a median of 12 days (IQR 9–20), with 75% of events occurring by POD 19. A summary of all 29 cases is provided in Table 1. The clinical monitoring and diagnostic parameters used to detect and confirm pedicle compromise in each reviewed case are summarized separately in Table 2. Surveillance relied predominantly on clinical examination, which was the reported monitoring method in all 29 cases and the sole modality in the majority. Handheld, implantable, or external Doppler was documented in nine cases, while adjunctive imaging or objective perfusion assessment—indocyanine green angiography, formal angiography or angio‐CT, duplex ultrasonography, intraoperative fluorescein, or tissue oximetry—was reported in only a minority. The compromise itself was most often heralded by frank clinical events, chiefly anastomotic or wound hemorrhage and progressive flap discoloration, rather than by a pre‐symptomatic monitoring alarm. This heterogeneity, and the frequent reliance on clinical examination alone without objective confirmation of vascular status, both limits cross‐study comparison and underscores that the diagnosis of pedicle compromise across this literature is itself largely presumptive.

**TABLE 1:** Literature review: Free flap survival despite pedicle compromise—summary of 29 cases.

Author, year	Flap type	Recipient site	Age/sex	Pre‐XRT	POD of compromise	Vessel(s) involved	Management strategy	Anticoagulation	Outcome (% flap survival)
Rath (1986)	Musculocutaneous (LD)	Left tibia	28/M	No	POD 17	Posterior tibial artery	Operative: tourniquet, ligation, no re‐anastomosis	NR	~95% survival; minimal necrosis
Keen et al. (1987)	Jejunal	Pharynx/cervical esophagus	68/M	Yes	POD 19	Vascular pedicle (transected)	Operative: jejunostomy, esophagostoma; fistula closed	NR	~95%; swallowing restored
Jones et al., (1994)	Jejunal	Pharyngoesophageal	68/M	No	POD 12	Mesenteric artery (saphenous vein graft)	Operative: exploration, necrotic area resected, fistula	NR	Yes (2 cm necrotic area); patient died of pneumonia at 3 months
Skrbic (1995)	Fasciocutaneous (radial forearm)	Bilateral heels	37/M	No	POD 12	Radial → posterior tibial artery	Operative: exploration, ligation	NR (dextran given as prophylaxis only)	100%; healthy flaps at discharge
Van Landuyt et al. (1996) (Case 1)	Muscle + STSG (LD)	Midfoot	69/F	No	~POD 150	Arterial bypass	Conservative: patient declined reintervention	NR	~70%; ambulated until unrelated death at 8 months
Van Landuyt et al. (1996) (Case 2)	Muscle + STSG (rectus)	Forefoot	56/M	No	~POD 90	Arterial bypass + flap pedicle	Conservative: no reintervention	NR	100%; full ambulation at 1.5 years
Van Landuyt et al. (1996) (Case 3)	Muscle + STSG (rectus)	Forefoot	68/M	No	~POD 240	Arterial bypass (chronic occlusion)	Conservative: no reintervention	NR	100%; walking without claudication
Salgado et al. (2002) (Case 1)	Myocutaneous (TRAM)	Right breast	48/F	No	POD 100	Vascular pedicle (artery & vein)	Conservative: observation	NR	66%; partial central loss, healed secondarily
Salgado et al. (2002) (Case 2)	Osteocutaneous (fibula)	Mandible/FOM	62/M	No	POD 8	NR (anastomotic bleed)	Operative: NR	NR	100%
Salgado et al. (2002) (Case 3)	Osteocutaneous (fibula)	Mandible	38/M	No	POD 10	NR (anastomotic bleed)	Operative: NR	NR	100%
Salgado et al. (2002) (Case 4)	Fasciocutaneous (radial forearm)	Right plantar foot	66/M	No	POD 166	Bypass vessel (occluded)	Conservative: observation, NPWT	LMWH (dose/duration NR)	100%
Salgado et al. (2002) (Case 5)	Osteocutaneous (fibula)	Mandible/FOM	61/M	No	POD 13	NR (anastomotic bleed)	Operative: NR	NR	100%
Salgado et al. (2002) (Case 6)	Osteocutaneous (fibula)	Mandible/FOM	47/M	Yes	POD 20	NR (anastomotic bleed)	Operative: NR	NR	100%
Chen (2002)	Jejunal	Pharynx/cervical esophagus	37/F	No	POD 17	Transverse cervical artery, ext. jugular vein	Operative: observation, necrotic portion excised, stoma	NR	~80%; oral feeding resumed at 76 days
Kissun et al. (2004)	Musculocutaneous (LD)	Cervical region	30/F	Yes	POD 6	External carotid → common carotid (ligated)	Operative: carotid repair, then ligation (POD 6)	NR	100%; fistula closed; no neurological deficit
Burns et al. (2005) (Case 1)	Fasciocutaneous (radial forearm)	Tongue (right)	59/F	NR	POD 19	Internal jugular vein	Operative: hematoma evacuation, IJV ligation	NR	100%; alive and disease‐free at 5 years
Burns et al. (2005) (Case 2)	Fasciocutaneous (radial forearm)	Tongue (right)	69/M	NR	POD 11	Internal jugular vein	Operative: IJV repair (POD 6), then ligation	NR	100%; alive at 21 months
Burns et al. (2005) (Case 3)	Fasciocutaneous (radial forearm)	Tongue (left)	49/F	NR	POD 6	Internal jugular vein, facial artery	Operative: pedicle division and ligation	NR	100%; alive and well at 2 years
Godden et al. (2005)	Fasciocutaneous (radial forearm)	Tongue	40/F	NR	POD 9	Superior thyroid artery, internal jugular vein	Conservative: local hemostasis; no re‐anastomosis	NR	100%; flap well‐perfused at follow‐up
Gurunluoglu (2010)	Replantation (thumb)	Thumb	NR/M	No	POD 12	Arterial anastomosis (thrombosis)	Conservative: observation only	Aspirin (daily, continued)	100%; functional and sensate at 5 months
Enajat et al. (2010)	Fasciocutaneous (SIEA)	Breast	64/F	Yes	POD 12	Internal mammary artery and vein	Operative: hematoma evacuation, re‐anastomosis × 2, then ligation	Heparin (post re‐anastomosis; dose/duration NR)	75%; 25% fat necrosis superior pole; flap viable
Wise et al. (2011)	Fasciocutaneous (ALT)	Neck/floor of mouth/tongue base	60/M	Yes	POD 9	Facial artery, external jugular vein	Operative: exploration, vessel ligation (friable walls)	NR	100%; flap viable; patient had repeat XRT
Adani et al. (2013)	Replantation (wrap‐around great toe)	Thumb	32/M	No	POD 7	First dorsal metatarsal artery and vein	Conservative (after failed re‐exploration): IV heparin + dextran	LMWH + dextran (inpatient); aspirin 300 mg × 7 days	~95%; small distal necrosis; nail grew normally at 10 months
Granzow et al. (2015) (Case 1)	Osteocutaneous (fibula)	Right mandible	76/F	No	POD 17	Peroneal → superior thyroid artery	Operative: two revisions (POD 4, 6), then ligation	NR	100%; complete healing at 4.5 years
Granzow et al. (2015) (Case 2)	Osteocutaneous (fibula)	Right posterior mandible	39/F	No	POD 11	Arterial and venous pedicle (thrombosis)	Operative: debridement, supraclavicular flap coverage	NR	100%; intraoral flap viable
Granzow et al. (2015) (Case 3)	Muscle (gracilis)	Right ankle	61/F	No	POD 10	Anterior tibial artery	Operative: two revisions (POD 7, 9), then ligation	IV heparin (continuous drip, restarted post‐POD 7)	~95%; minor necrosis over pedicle; flap largely preserved at 4 years
Pototschnig et al. (2016)	Fasciocutaneous (ALT)	Medial dorsal foot	25/M	No	POD 6	Pedicle artery (+venous congestion)	Conservative: flap left in place despite occlusion	LMWH (daily from admission; AT deficiency diagnosed post‐op)	~95%; epidermolysis only; survived at ≥ 12 months
Strübing et al. (2018)	Fasciocutaneous (parascapular)	Proximal tibia/knee	55/M	No	POD 49	AV‐loop (saphenous vein graft, superficial femoral artery)	Conservative: anticoagulation + prostaglandin therapy	LMWH 0.6 mL bid × 7 days; PGE1 40 μg bid IV	100%; fully integrated at 11 weeks; returned to work at 36 weeks
Vanmierlo et al. (2020)	Replantation (digital)	Right middle finger	87/M	No	34 years post‐replantation	Radial digital artery (atherosclerotic occlusion)	Conservative: local wound care, analgesics	NR	100%; ulceration healed at 3 months

Abbreviations: ALT, anterolateral thigh; AT, antithrombin; AV, arteriovenous; bid, twice daily; FOM, floor of mouth; IJV, internal jugular vein; LD, latissimus dorsi; LMWH, low molecular weight heparin; NPWT, negative pressure wound therapy; NR, not reported; PGE1, prostaglandin E1; POD, postoperative day; SCC, squamous cell carcinoma; SIEA, superficial inferior epigastric artery; STSG, split‐thickness skin graft; TRAM, transverse rectus abdominis myocutaneous; XRT, radiotherapy.

**TABLE 2:** Clinical monitoring and diagnostic parameters used to detect and confirm pedicle compromise across the 29 reviewed cases.

Author, year	POD of compromise	Monitoring/assessment modality	Mode of detection and presentation of compromise	Objective vascular confirmation
Rath (1986)	POD 17	Clinical examination	Massive acute bleeding from ruptured septic pseudo‐aneurysm	None (clinical only)
Keen et al. (1987)	POD 19	Clinical examination; endoscopy	Dysphagia, no flow through distal jejunal segment, mucosal edema, small area of necrosis	Endoscopy
Jones et al. (1994)	POD 12	Intraoperative fluorescein; Wood's lamp; visual inspection; endoscopy	Hypotension, unresponsiveness, large right‐neck haematoma	Fluorescein; endoscopy
Skrbic (1995)	POD 12	Clinical examination	Massive bleeding from left retromalleolar scar during physiotherapy	None (clinical only)
Van Landuyt et al. (1996) (Case 1)	~POD 150	Clinical examination	Flap sloughing and ulceration following bypass occlusion	None (clinical only)
Van Landuyt et al. (1996) (Case 2)	~POD 90	Angiography	Acute occlusion of bypass and flap artery	Angiography
Van Landuyt et al. (1996) (Case 3)	~POD 240	Duplex ultrasound + Doppler	No pulse in bypass or flap pedicle	Duplex ultrasound
Salgado et al. (2002) (Case 1)	POD 100	Clinical examination	Central necrosis of TRAM flap	None (clinical only)
Salgado et al. (2002) (Case 2)	POD 8	Clinical examination	Bleeding/hematoma from anastomosis	None (clinical only)
Salgado et al. (2002) (Case 3)	POD 10	Clinical examination	Bleeding/hematoma from anastomosis	None (clinical only)
Salgado et al. (2002) (Case 4)	POD 166	Clinical examination	Flap congestion with risk of eventual necrosis	None (clinical only)
Salgado et al. (2002) (Case 5)	POD 13	Clinical examination	Hematoma and bleeding from anastomosis	None (clinical only)
Salgado et al. (2002) (Case 6)	POD 20	Clinical examination	Anastomotic bleed	None (clinical only)
Chen (2002)	POD 17	Clinical examination; barium swallow	Dusky discoloration of distal external portion	Barium swallow
Kissun et al. (2004)	POD 6	Clinical examination	Acute hemorrhage from neck incision (Day 4 and Day 6); bleeding from flap muscle	None (clinical only)
Burns et al. (2005) (Case 1)	POD 19	Clinical examination	Fever, rigors, bleeding from drain sites, neck swelling	None (clinical only)
Burns et al. (2005) (Case 2)	POD 11	Clinical examination	Bleeding from tracheostomy and mouth	None (clinical only)
Burns et al. (2005) (Case 3)	POD 6	Clinical examination	Brisk bleeding from mouth	None (clinical only)
Godden et al. (2005)	POD 9	Clinical examination	Neck infection; dramatic arterial hemorrhage from neck with pedicle necrosis	None (clinical only)
Gurunluoglu (2010)	POD 12	Clinical examination; Doppler	Loss of Doppler signal, sluggish capillary refill	Doppler
Enajat et al. (2010)	POD 12	Clinical examination; implantable and external Doppler	Abnormal stretch sensation, acute breast swelling, pale‐blue flap, delayed capillary refill; later swelling and superficial blisters	Implantable/external Doppler
Wise et al. (2011)	POD 9	Clinical examination; Doppler	Neck abscess with thrombosis of both artery and vein; no Doppler signals	Doppler
Adani et al. (2013)	POD 7	Clinical examination (capillary refill, pinprick); Doppler	No capillary refill, no bleeding on pinprick, no Doppler signal	Doppler
Granzow et al. (2015) (Case 1)	POD 17	Clinical examination; Doppler; ViOptix tissue oximetry	Recurrent arterial anastomosis avulsion and hematoma	Doppler; tissue oximetry
Granzow et al. (2015) (Case 2)	POD 11	Clinical examination; Doppler	Neck skin necrosis, salivary collection, thrombosed pedicle	Doppler
Granzow et al. (2015) (Case 3)	POD 10	Clinical examination; Doppler	Repeated loss of Doppler signal, arterial thrombosis	Doppler
Pototschnig et al. (2016)	POD 6	Clinical examination + angio‐CT	Flap congestion, bluish discoloration; angio‐CT confirmed arterial occlusion	Angio‐CT
Strübing et al. (2018)	POD 49	Clinical examination + indocyanine green (ICG)	Mottled, partially bluish, cold flap	ICG angiography
Vanmierlo et al. (2020)	34 years post‐replantation	Clinical examination	Ischemic ulcer, cyanosis, delayed capillary refill (> 5 s)	None (clinical only)

Abbreviations: angio‐CT, computed tomography angiography; ICG, indocyanine green; POD, postoperative day; TRAM, transverse rectus abdominis myocutaneous.

Stratified analyses found no statistically significant differences in autonomization timing by flap composition (*p* = 0.28, Kruskal–Wallis test), recipient site (*p* = 0.21), prior radiotherapy (*p* = 0.49), gender (*p* = 0.68), or comorbidities (*p* = 0.80). However, patients with very early autonomization (POD ≤ 7, *n* = 4) were significantly younger than those with later compromise (median 31 vs. 60 years; *p* = 0.01). A moderate positive Spearman correlation was found between patient age and POD of definitive pedicle non‐viability (ρ = 0.42, *p* = 0.03, *n* = 27). Regarding management, flaps managed conservatively showed a higher median POD compared with those managed operatively (90 days [IQR 12–150] vs. 12 days [IQR 9.5–17]; *p* = 0.03), reflecting a selection effect rather than evidence of treatment superiority: later‐onset compromise is less likely to trigger operative re‐exploration.

Younger patients in our analysis showed earlier autonomization. This aligns with established evidence that aging reduces angiogenic capacity via impaired endothelial progenitor cell function and diminished hypoxia‐inducible factor signaling (Hoenig et al. 2008; Wüster et al. 2026), though a direct clinical correlation in the free flap setting has not been previously reported (Mücke et al. 2012; Wüster et al. 2026).

Indocyanine green angiography is a valuable adjunct for assessing tissue perfusion in free flap surgery. By visualizing real‐time blood flow, ICG allows surgeons to detect areas of compromised perfusion, guiding both intraoperative and postoperative management decisions (Choudhary et al. 2023; Adelsberger et al. 2019; Ludolph, Horch, et al. 2019). In Case 1, bedside Fluobeam ICG imaging identified peripheral vascularization distant from the pedicle insertion site at the time of clinical ischemia; this finding supported the decision to observe rather than re‐explore. In Case 2, ICG monitoring at the same timepoint showed marginal fluorescence consistent with peripheral neovascularization, but the image files were not retained.

It must be emphasized that ICG angiography should be interpreted in conjunction with clinical and Doppler findings rather than in isolation. The dynamic video sequence provided sequential perfusion data that clarified the spatial origin and direction of flow, supporting the clinical interpretation. In both cases, the decision to observe rested on serial Doppler recordings, clinical signs, and ICG perfusion pattern together, not on imaging alone.

The decision to observe rather than re‐explore was not arbitrary; in both patients it followed an explicit, prospectively applied set of criteria that we regard as the minimum threshold for even considering conservative management. Observation was entertained only when all of the following conditions were met simultaneously. First, the compromise had to occur beyond the early postoperative window—here postoperative day 6 in both cases—a timepoint at which peripheral autonomization from the recipient bed may plausibly be underway, rather than within the first 72 h when the flap remains wholly pedicle‐dependent. Second, there had to be concordant, multimodal evidence of established peripheral perfusion arising independently of the pedicle: viable pink flap margins in direct contact with the wound bed contrasting with central pallor, preserved non‐pulsatile Doppler signal confined to the flap periphery, and—where obtainable—indocyanine green fluorescence originating from the flap margin distant from the pedicle axis rather than propagating back along it. Third, there had to be a complete and unambiguous loss of pedicle‐derived arterial signal, so that the clinical question was pedicle‐independent survival rather than partial or salvageable pedicle flow. Fourth, there had to be no competing indication mandating immediate return to theater—no venous congestion, no progressive or full‐thickness necrosis, no active hemorrhage, no infection, and no systemic instability. Finally, conservative management was permissible only in a setting of continuous bedside monitoring with immediate operative capability, so that re‐exploration could be undertaken without delay should any parameter deteriorate. Both patients satisfied every one of these conditions, which is precisely why they were judged suitable for observation; a case failing any single criterion would have been returned to the operating room. This framework is presented not as a validated selection protocol but as a transparent account of the reasoning that governed these two specific decisions, so that the basis for departing from the standard of immediate re‐exploration is explicit and reproducible.

Just as important as defining who may be observed is defining when observation must stop. In both patients we applied an explicit set of prospectively agreed thresholds any one of which mandated immediate return to theater and overrode the decision to observe. These were: (1) new, expanding, or full‐thickness necrosis, or any increase in the extent of dermal compromise on serial examination; (2) loss or regression of the peripheral perfusion signal on which the decision rested, whether the marginal Doppler signal or the marginal indocyanine green fluorescence; (3) active anastomotic or wound hemorrhage, or an expanding haematoma; and (4) any systemic instability. Because the penalty for persisting with observation in a flap that has not autonomized is total and irreversible loss, these thresholds were set deliberately low, and the appearance of any single one would have ended observation in favor of surgical re‐exploration.

Both cases presented here offer specific contributions to the existing literature. First, they represent presumptive pedicle‐independent fasciocutaneous flap survival at postoperative day 6, among the earliest timepoints described in the clinical literature (Yoon and Jones 2016). Second, both involve lower extremity recipient sites, which are substantially underrepresented in the published literature relative to head and neck cases, and which present additional reconstructive challenges including dependent positioning and limited recipient vessel caliber. Third, both cases employed real‐time bedside ICG monitoring using the Fluobeam system to guide conservative decision‐making—representing a specific postoperative application that is not widely adopted in the context of suspected pedicle compromise.

This report has several important limitations. First, the diagnosis of pedicle compromise in both cases is presumptive rather than confirmed. No angiographic confirmation of vascular compromise was obtained in either patient—neither catheter angiography, CT, or CT‐angiography, nor operative inspection of the anastomosis was performed—because the very decision under evaluation was to withhold intervention, and any confirmatory maneuver would either have required the re‐exploration we were seeking to avoid or exposed a stable patient to additional risk and contrast load. The diagnosis therefore rested on indirect evidence: complete loss of pedicle‐derived Doppler signal together with overt clinical ischemia, supported by the indocyanine green perfusion pattern. While this combination is strongly suggestive of pedicle failure, it does not carry the diagnostic certainty of direct angiographic or intraoperative visualization. Two consequences follow. On the one hand, the possibility that residual subclinical antegrade pedicle flow—below the detection threshold of handheld Doppler—contributed to flap survival cannot be formally excluded, so the true perfusion state of the pedicle at the moment of presumed compromise remains unproven. On the other, these cases cannot establish that a completely and definitively thrombosed pedicle was survived. What can be stated with confidence is narrower: the flaps survived despite loss of the conventional signals used to monitor pedicle patency. Any inference regarding pedicle‐independent survival must be read against this diagnostic uncertainty, and the same caveat applies with equal or greater force to the reviewed literature, in which objective confirmation of vascular status was likewise frequently absent. Second, the systematic literature review on which the contextual analysis is based is composed entirely of case reports and series, with inherent publication bias strongly favoring successful outcomes. There is no comparator group of failed conservative management cases—cases where observation was attempted and the flap was subsequently lost are systematically underreported, and this represents the most critical source of selection bias. This absence of failure data precludes causal inference and means that the true failure rate of conservative management cannot be estimated from available evidence, not because such failures do not occur, but because negative outcomes in this setting are not reported. Third, all subgroup analyses from the literature review must be interpreted cautiously given the small sample sizes. Fourth, the lack of a universally accepted definition of flap autonomization limits cross‐study comparisons.

The observation that conservative management was associated with later‐onset compromise reflects a selection effect rather than treatment efficacy, and this association must not be interpreted as evidence that conservative management is safer or more effective than operative intervention. Delayed re‐exploration carries the real risk of complete flap loss if autonomization has not occurred. This danger deserves to be stated in concrete terms, because it is the principal hazard of the approach described here. When a flap has not yet autonomized, its survival remains entirely dependent on pedicle inflow, and a decision to observe rather than re‐explore allows ischemia to progress unchecked toward irreversible necrosis. Salvage is time‐critical: reported flap‐salvage rates decline steeply with every hour that elapses between the detection of compromise and operative intervention, and beyond a narrow window, successful thrombectomy or re‐anastomosis is no longer achievable (Kamali et al. 2021; Gupta et al. 2024). A conservative decision that proves incorrect therefore does not merely delay salvage—it forfeits it, converting a potentially recoverable flap into a certain and total loss. This asymmetry, in which the penalty for an incorrect conservative decision is far greater than that for an unnecessary re‐exploration, is the reason the threshold to abandon observation in favor of surgery must remain deliberately low.

When multimodal monitoring—including complete absence of pedicle‐derived Doppler signal, clinical ischemia, and ICG imaging demonstrating stable peripheral perfusion—is concordant, selective conservative management may be a reasonable consideration, particularly in younger patients or when compromise occurs beyond postoperative day 6. However, this approach should be undertaken with extreme caution. The standard of care remains immediate surgical re‐exploration upon detection of vascular compromise, and published evidence consistently demonstrates that salvage rates decline substantially with increasing delay from detection to operative intervention (Kamali et al. 2021; Gupta et al. 2024). Conservative management should therefore only be considered when there is unambiguous, multimodal, and serially confirmed evidence of established peripheral perfusion—and even then requires continuous monitoring capability and immediate access to operative intervention should the clinical picture deteriorate. It must not be generalized as a standard of care. In addition, systemic anticoagulation with unfractionated heparin may be considered as an adjunct to limit thrombus propagation in cases where conservative management is elected, although its specific role in this setting has not been prospectively evaluated and must be weighed against the individual patient's bleeding risk. Prospective multicenter studies with standardized monitoring protocols and systematic capture of both successful and failed conservative management outcomes are needed before formal practice recommendations can be established.

Several specific gaps in the existing literature warrant targeted investigation. First, there are currently no published criteria for selecting candidates for conservative management of suspected pedicle compromise—no consensus statement endorses this approach, and published guidance uniformly recommends immediate re‐exploration (Dort et al. 2017). Second, standardized postoperative monitoring protocols do not exist: the Enhanced Recovery After Surgery Society has explicitly acknowledged that no universally acceptable monitoring approach has been defined (Dort et al. 2017), and recent systematic reviews confirm wide heterogeneity in the use and interpretation of adjunctive technologies including ICG angiography. Indocyanine green angiography is a qualitative, at best semi‐quantitative, modality: fluorescence intensity varies with dye dose, injection timing, camera gain and working distance, and ambient lighting, and no validated threshold separates pedicle‐derived from neovascular perfusion, so interpretation is operator‐dependent and subject to interobserver variability. The signal is generated predominantly from the superficial dermal and subdermal plexus, and the limited near‐infrared tissue penetration of the technique means that deep or pedicle‐level flow can be neither confirmed nor excluded (Lacey et al. 2023; Poen et al. 2026).

Future prospective studies should adopt pre‐specified multimodal monitoring protocols—including quantitative serial Doppler recording and dynamic ICG perfusion sequences—and should define in advance the combination of findings that would justify observation rather than immediate operative intervention. Third, the influence of patient‐specific factors on early autonomization capacity remains poorly characterized in the human clinical literature despite strong experimental precedent (Clarke et al. 1985; Cordeiro et al. 1999); prospective registries should stratify by these variables from the outset. Finally, future registries must systematically capture failed conservative management outcomes, as the current literature is composed almost entirely of successes, making it impossible to estimate the true risk of this approach.

## Author Contributions

**Filippo A. G. Perozzo:** conceptualization, literature review, data analysis, writing, correspondence. **Diwakar Phuyal**, **Julia Ting**, and **James Owens Gaston:** literature review, data collection, manuscript review and editing. **L. Scott Levin:** supervision, manuscript review and editing. **Alberto Franchi:** conceptualization, supervision, manuscript review and editing.

## Funding

The authors have nothing to report.

## Ethics Statement

The study was performed in accordance with the principles of the Declaration of Helsinki.

## Consent

The patients provided written informed consent for the publication and use of their images.

## Conflicts of Interest

The authors declare no conflicts of interest.

## Supporting information


**Figure S1:** PRISMA flowchart summarizing the study identification, screening, eligibility assessment, and inclusion process for the review.

## Data Availability

The data that support the findings of this study are available from the corresponding author upon reasonable request.
