# Supplementary material for: Conservative Management of Presumptive Free Flap Pedicle Compromise on Postoperative Day 6: Report of Two Cases and Literature Review
Source: Microsurgery. 2026 Oct 31;46(8):e70314. doi: 10.1002/micr.70314 (PMC13722995; doi:10.1002/micr.70314)
Supplement: Supplementary file 1 [file MICR-46-e70314-s001.docx]

Records identified through database searching
(n = 2357)

**Screening**

**Included**

**Eligibility**

**Identification**

Records after duplicates removed
(n = 513)

Records screened
(n = 1844)

Records excluded (n = 1744)

Full-text articles assessed for eligibility
(n = 100)

Studies excluded (n = 82)

Non-microsurgical reconstruction (n = 17)

Non-English language (n = 13)

Isolated venous thrombosis (n = 15)

Intraoperative thrombosis (n = 4)

No flap outcome details (n = 22)

Insufficient clinical details (n = 11)

Studies included in quantitative synthesis
(n = 18)

**Supplementary Figure 1**. PRISMA flowchart summarizing the study identification, screening, eligibility assessment, and inclusion process for the review.
